# The Cooperative Landscape of Multinational Clinical Trials

**DOI:** 10.1371/journal.pone.0130930

**Published:** 2015-06-23

**Authors:** David Hsiehchen, Magdalena Espinoza, Antony Hsieh

**Affiliations:** 1 Mount Auburn Hospital, Cambridge, Massachusetts 02138, United States of America; 2 Northwestern Memorial Hospital, Chicago, Illinois 60611, United States of America; Mario Negri Institute for Pharmacology Research, ITALY

## Abstract

The scale and nature of cooperative efforts spanning geopolitical borders in clinical research have not been elucidated to date. In a cross-sectional study of 110,428 interventional trials registered in Clinicaltrials.gov, we characterized the evolution, trial demographics, and network properties of multinational clinical research. We reveal that the relative growth of international collaboratives has remained stagnant in the last two decades, although clinical trials have evolved to become much larger in scale. Multinational clinical trials are also characterized by higher patient enrollments, industry funding, and specific clinical disciplines including oncology and infectious disease. Network analyses demonstrate temporal shifts in collaboration patterns between countries and world regions, with developing nations now collaborating more within themselves, although Europe remains the dominant contributor to multinational clinical trials worldwide. Performances in network centrality measures also highlight the differential contribution of nations in the global research network. A city-level clinical trial network analysis further demonstrates how collaborative ties decline with physical distance. This study clarifies evolving themes and highlights potential growth mechanisms and barriers in multinational clinical trials, which may be useful in evaluating the role of national and local policies in organizing transborder efforts in clinical endeavors.

## Introduction

Large teams involving international collaborations are a growing theme across many research disciplines and are increasingly associated with high impact science [1–4]. The ability of international collaboratives to harness human capital, material resources, and infrastructure to achieve otherwise prohibitive achievements has been highlighted in recent examples including the comprehensive annotation of functional DNA elements, discovery of the Higgs boson, and landing of the Philae probe on a comet [5–7]. Notably, many seminal advances in medicine, including the recent demonstration of antiviral therapy as a modality to reduce HIV transmission, the role of computed tomography in lung cancer screening, superior outcomes after coronary-artery bypass grafting in diabetic patients, and the efficacy of the immunomodulatory antibody ipilimumuab in metastatic melanoma, have also stemmed from large multinational research teams [8–11].

Past studies characterizing authorship dynamics of research publications have revealed generic structures of collaborative networks and properties of team assembly mechanisms, though unique traits exist across subject disciplines [4, 12–14]. Nonetheless, to date, the scale and nature of cooperative efforts in clinical research involving human subjects spanning political and geographic borders have not been comprehensively elucidated. Herein, we characterized the evolution, trial demographics, network properties, and geographic constraints of multinational clinical research, defined as interventional trials with clinical sites enrolling patients in more than one country. Importantly, our criteria for multinational clinical trials does not rely on authorship affiliations, the conventional method of analyzing collaborative ties, given the high incidence of non-publication in clinical trials and the likely involvement of international authors that are not directly involved in patient recruitment [15].

## Materials and Methods

**Fig 1:**
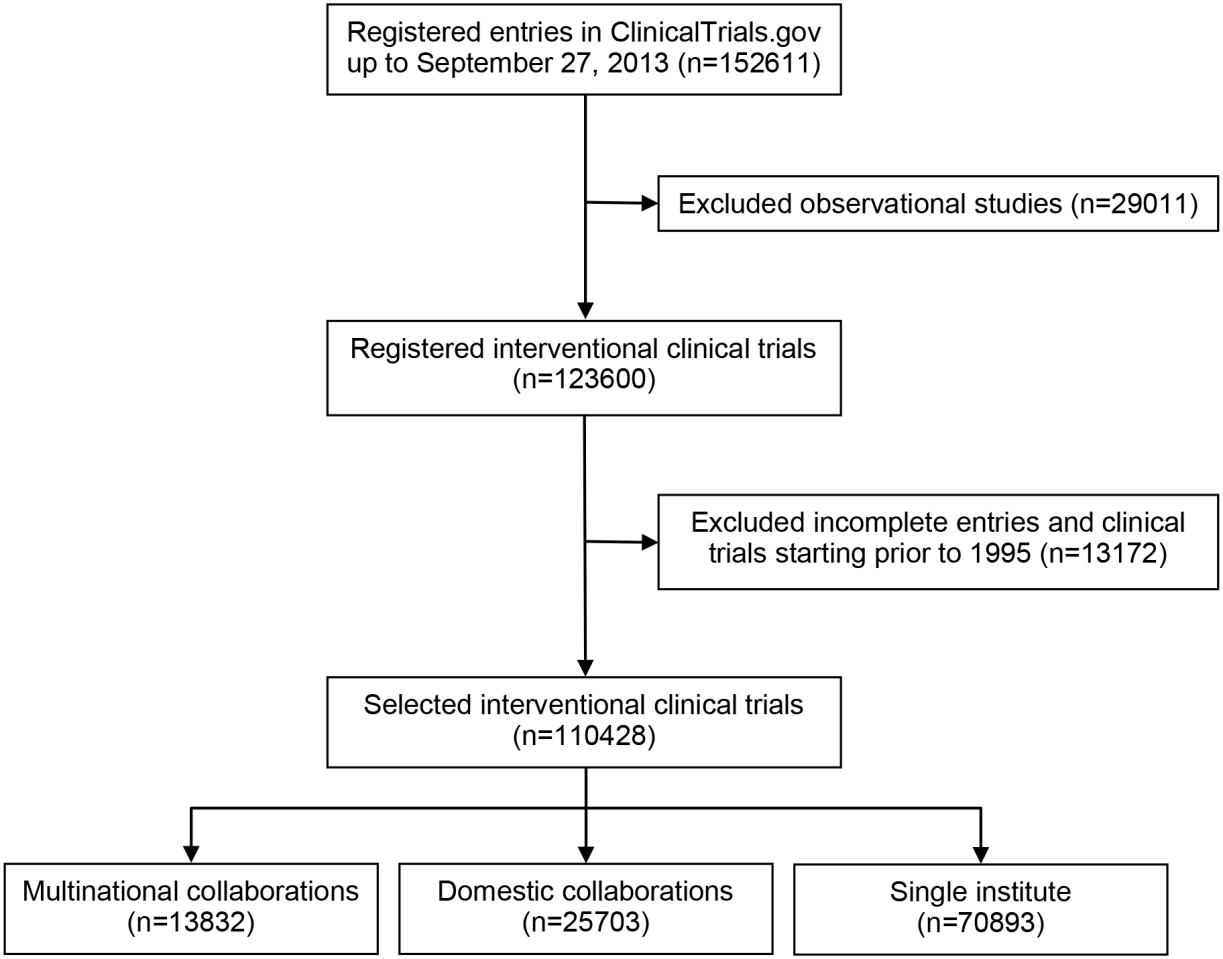
The selection of clinical trials from ClinicalTrials.gov.

We downloaded the entire Aggregate Analysis of ClinicalTrials.gov database (updated on September 27, 2013) including data for 152,611 clinical trials from the Clinical Trials Transformation Initiative portal (http://www.ctti-clinicaltrials.org). We focused on clinical trials with start years from 1995 and onwards and included only interventional trials (identified through the *study type* field) without missing data regarding the location of clinical sites enrolling patients (hereafter referred to as an institute), yielding 110,428 studies (Fig 1). While multiple clinical trial registries exist, we performed our analyses on ClinicalTrials.gov given its international scope, large scale, and accessibility.

To categorize clinical trials by clinical specialties, we examined Medical Subject Heading (MeSH) terms, vocabulary established by the National Library of Medicine (NLM) for indexing purposes, assigned to each clinical trial based on an NLM algorithm from the *condition browse* fields. MeSH terms encompass descriptors organized in a hierarchy, with broader headings including anatomical regions and diseases. By cataloguing MeSH terms by their broadest descriptors that corresponded to a clinical specialty, clinical trials could be assigned to the following systems: digestive, endocrine, cardiovascular, cancer, infectious, musculoskeletal, nervous, psychiatric, reproductive (created by combining male and female urogenital diseases), respiratory, and other (including integumentary, dental, congenital, and nutrition/metabolic).

Sites of patient enrollment for clinical trials were garnered from the *facilities* and *location countries* fields. Both nation-level and city-level networks were constructed, with either nations or cities acting as nodes and links between actors indicating a shared presence within the same clinical trial. Network analyses were performed using Visone [16]. Network-level metrics, such as density (number of edges divided by the number of possible edges) and average node degree, were determined using unweighted networks. Node-level metrics, including eigenvector, betweenness, and degree centrality, were determined using weighted networks with the value of links determined by the number of shared clinical trials between countries. The concepts of centrality measures are reviewed elsewhere, but briefly, degree centrality characterizes how well a node is connected to other nodes, eigenvector centrality characterizes how well connected a node is within a network particularly when linked to other nodes with high centrality, and betweenness centrality characterizes the degree to which a node lies on the shortest path between other nodes [17]. To determine whether physical distance may be associated with the likelihood of collaborative ties at the city-level, we utilized the Google Geocoding API to convert all cities into latitude and longitude coordinates. Distances between coordinates were then calculated using the great circle distance method.

## Results

**Fig 2:**
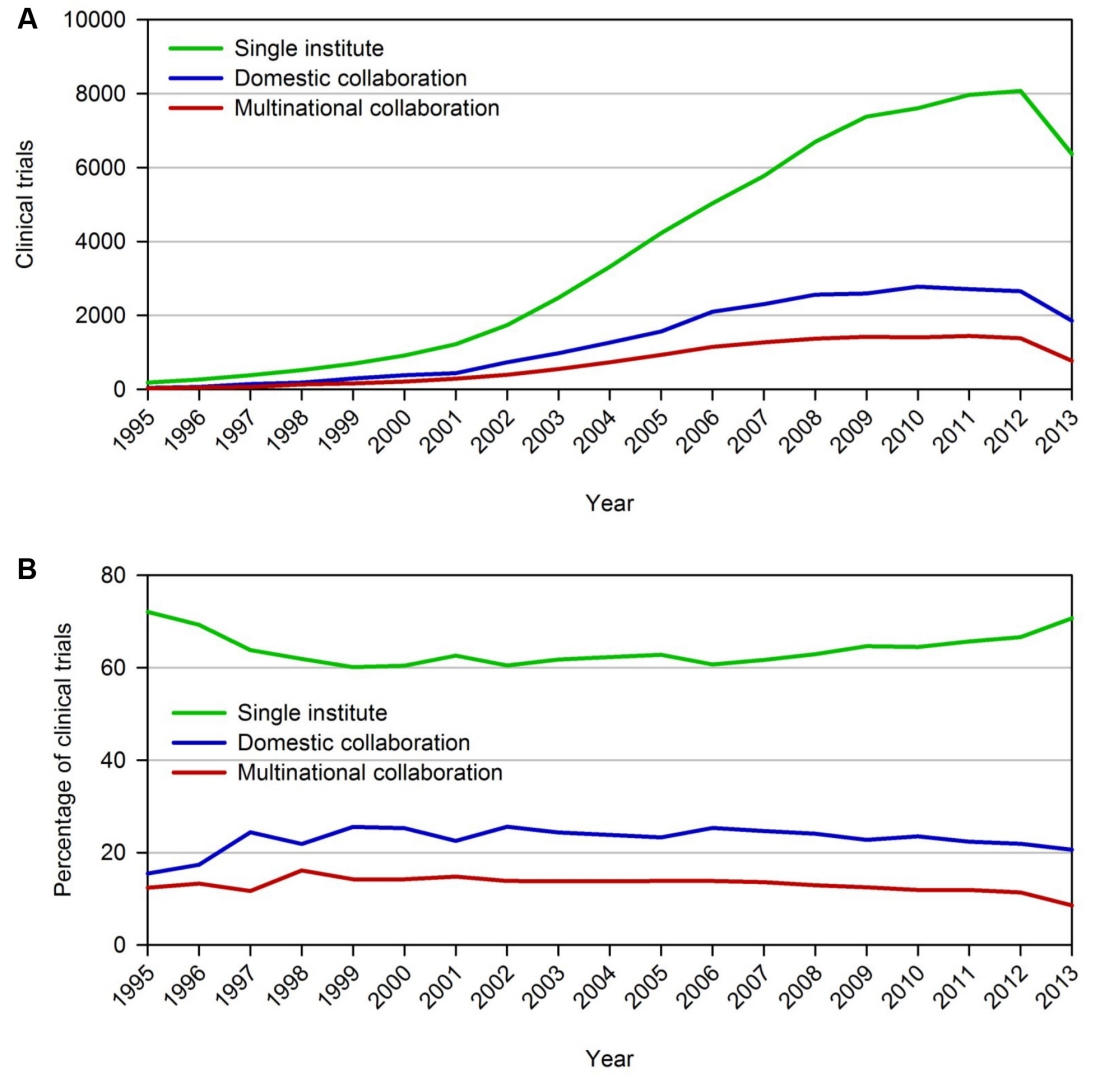
Growth of clinical trials. (A) Levels of clinical trials associated with single institutes, domestic collaborations, and multinational collaborations at specified starting years. The decline in trials in 2013 is due to the fact that the most recent database update from the Aggregate Analysis of Clinicaltrails.gov was from March of 2014 (which only included trials registered up until September 2013). (B) Data from Panel A as a percentage of all trials starting within the same year.

The number of registered trials attributed to either a single institute, domestic collaboration (composed of more than one institute within a country), or multinational collaboration (composed of at least two institutes each in a unique country) in ClinicalTrials.gov has grown considerably, particularly over the last decade (Fig 2A). However, in contrast to the increasing proportion of scholarly output credited to international collaborations across many research disciplines, relative levels of multinational clinical trials remained largely static (Fig 2B) [2, 6, 18].

**Fig 3:**
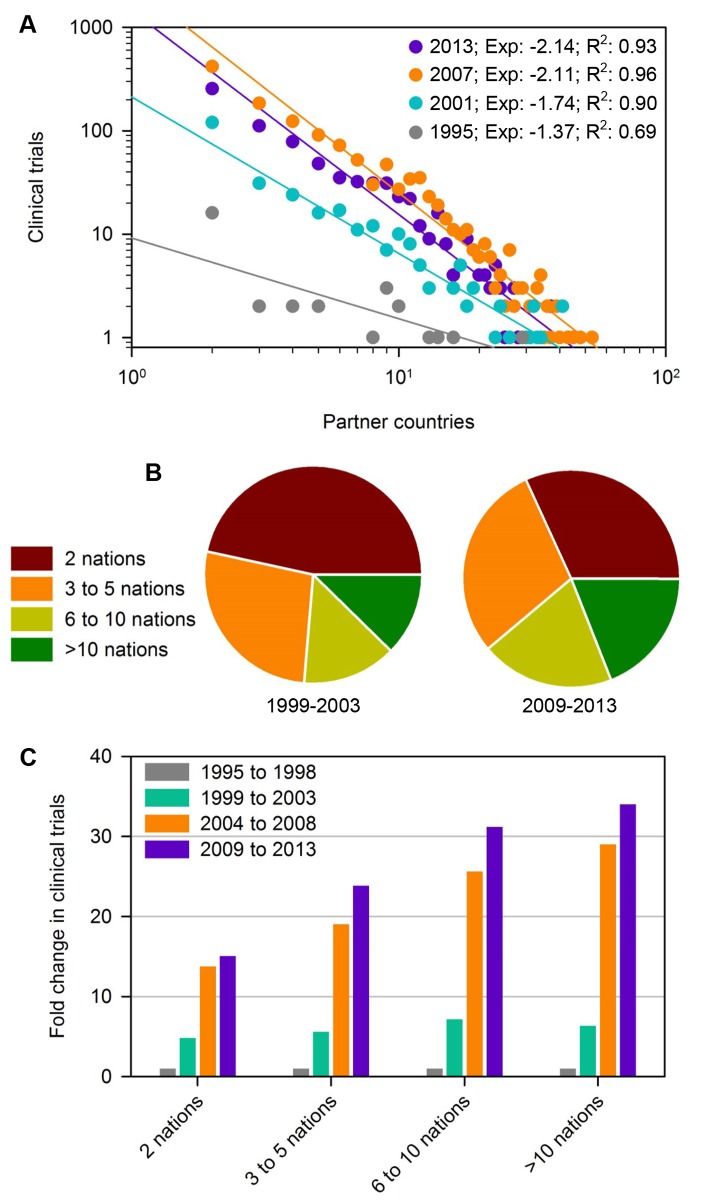
Shifts in the composition of clinical trial research teams over time. (A) The distribution frequency of unique country numbers associated with clinical trials. (B) Pie charts of trials categorized by the number of associated nations in two time periods, indicating the diminishing contribution of binary relations. (C) The relative fold increase in trials categorized by the number of associated nations over time relative to the time period from 1996 to 1998.

We next explored temporal changes in the constitution of multinational collaborations by examining the frequency distribution of unique partnering countries associated with clinical trials. Conspicuously, distribution frequencies for each year obeyed power-laws, with more recent years exhibiting decreased scaling exponents (Fig 3A). The slower power law decay of data from older years suggests that the distribution of unique partnering countries has become more equitable over time, i.e. the number of countries associated with trials has become less skewed with larger scale multinational clinical trials accounting for a growing fraction of all international collaboratives (Fig 3B). In fact, clinical trials involving at least ten nations were found to be the fastest growing category of clinical research (Fig 3C). Collectively, these results demonstrate a shift in the composition of clinical trial research teams.

**Fig 4:**
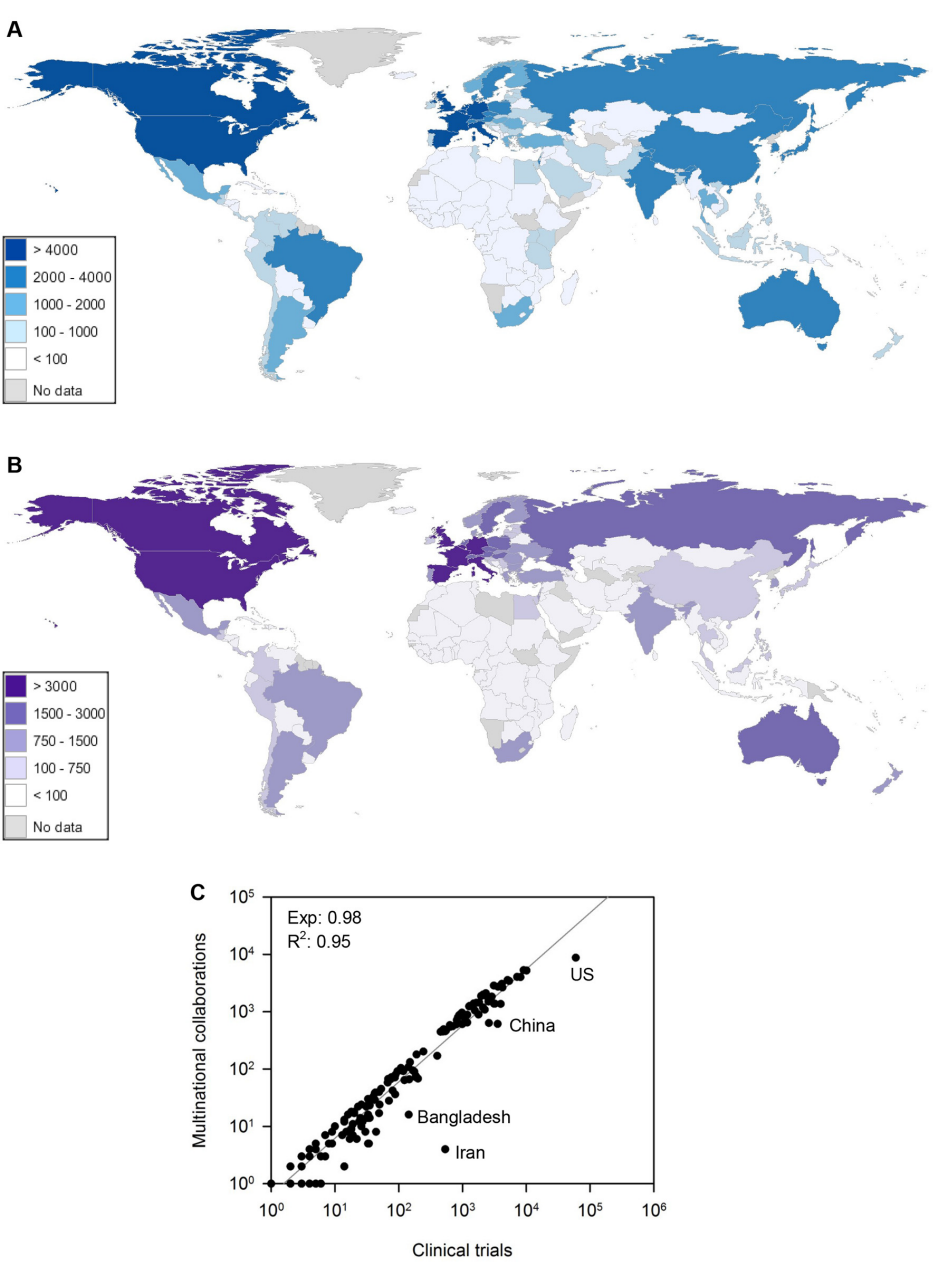
Nation-level production of trials. (A) The production of clinical trials by each nation worldwide. (B) The production of multinational clinical trials by each nation worldwide. Global maps were created using the freeware software StatPlanet (StatSilk). (C) Production of multinational trials by individual nations scales with total clinical trial output according to a power law with an exponent of approximately one, indicating isometric scaling. Each unit of analysis corresponds to a nation. Nations which deviate widely from the scaling phenomenon are labeled in the plot.

At the nation-level, the highest producers of multinational collaborations in clinical trials generally corresponded with the most prolific countries in clinical research (Fig 4A and 4B). Interestingly, multinational clinical trials scaled with total clinical trial output according to a linear power law known as isometry, which conventionally describes the constant proportionate growth of organismal body dimensions (Fig 4C). These findings suggest that a nation’s likelihood of participating in multinational clinical trials is predicted and bounded by a simple function of its research productivity. Isometric scaling was also recently shown to characterize urban indicators that typify individual consumption, suggesting that international collaboration in trials may serve as a resource utilized to sustain the accelerating growth of clinical research output among nations [19, 20]. Worldwide, isometric scaling described multinational clinical trial output with few exceptions, including the US, China, and Iran where rates of participation in multinational trials were less than predicted (Fig 4C).

**Fig 5:**
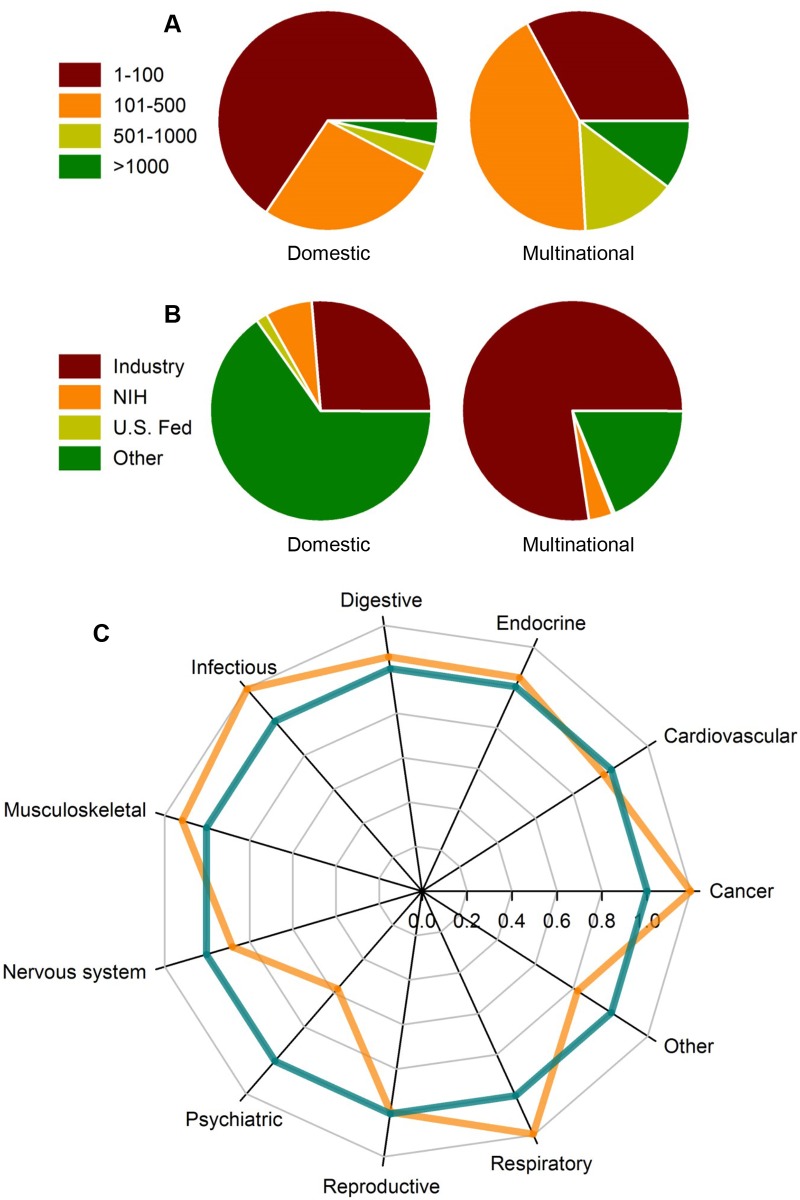
Characteristics of multinational clinical trials. (A) Pie charts of domestic and multinational trials categorized by patient enrollment size. (B) Pie charts of domestic and multinational trials categorized by funding. (C) A radar plot depicting relative levels of multinational trials (orange line) produced across different clinical disciplines. Relative levels were calculated as the ratio between the fraction of multinational trials in one subject field divided by all multinational trials and the fraction of trials in the same subject field divided by the total number of trials. The teal line denotes a ratio of one or when levels of multinational collaborations and total clinical trial output are relatively equal.

When we analyzed the properties of clinical trials involved in either multinational or domestic clinical trials, international collaborations were found to be associated with larger enrollment numbers and industry funding (Fig 5A and 5B). Multinational clinical trials were also more prevalent in certain clinical disciplines including oncology, pulmonology, and infectious disease (Fig 5C). On the other hand, clinical trials related to the nervous system, psychiatry, and cardiology were less likely to be involved in international collaborations.

**Fig 6:**
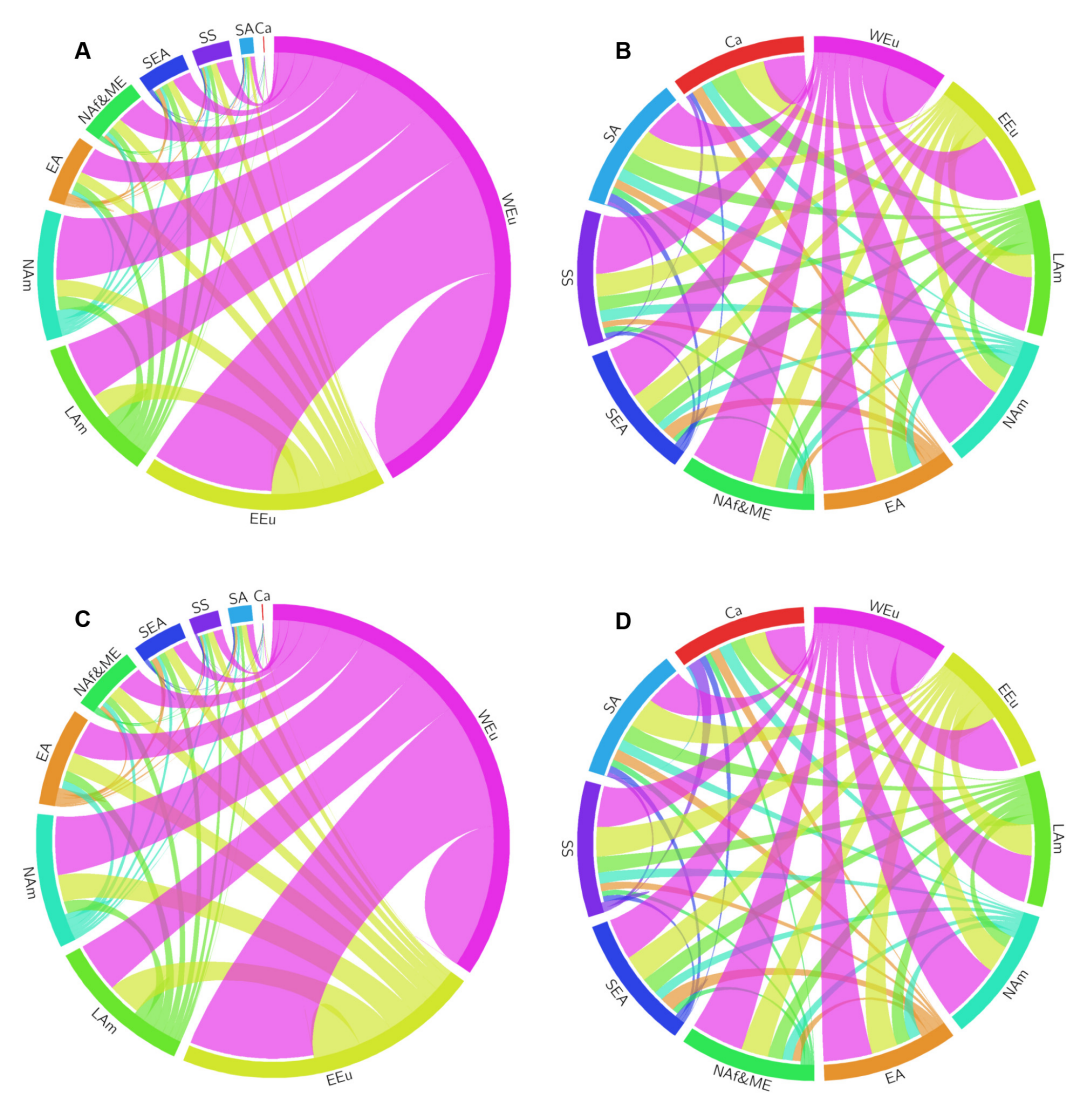
Collaborative ties in clinical trials among geopolitical regions. (A) and (B) Circular plots portray collaboration ties between countries participating in the most multinational trials from 1999 to 2003 and 2009 to 2013, respectively. The size of each country segment corresponds to the relative contribution of that country to all collaborations produced by the ten displayed nations. Tie widths at each end portray the relative contribution of collaborations between the two nations to all collaborations associated with each individual country. Tie values less than the 75th percentile were faded to aid visualization of strong ties. (C) and (D) Circular plots portray collaboration ties between groupings of nations by the UN human development index. VHHD, very high human development; HHD, high human development, MHD, medium human development; LHD, low human development.

To elucidate collaborative patterns between countries, we constructed weighted collaboration networks of clinical trials with nodes representing countries and tie weights representing the number of clinical trials shared between the two corresponding nations. Circular plots in Fig 6A and 6B illustrate how ties between the most collaborative countries have changed between 1999–2003 and 2009–2013, which demonstrates greater homogeneity in tie weights for the US over time and persistent idiosyncratic nation-nation relations [21]. For example, in the early 2000s, the strongest link among all select nations was between the USA and Canada, although within a decade ties between the USA and its multiple partners become more comparable in strength. Meanwhile, strong ties between Germany, France, Italy, and the United Kingdom endured over time.

**Fig 7:**
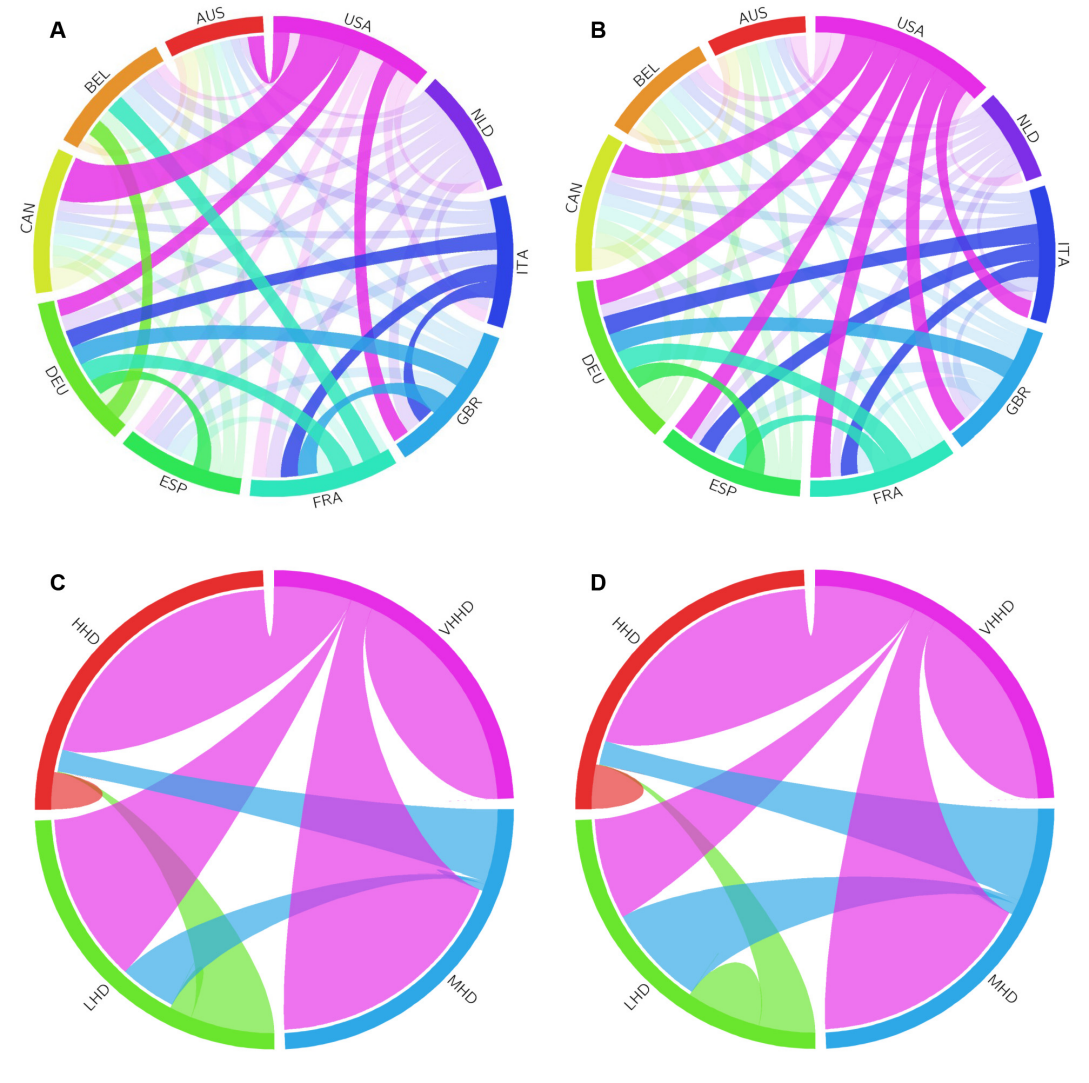
Circular plots of collaborations in trials among geopolitical regions. (A) A circular plot portrays absolute levels of collaborations in trials among geopolitical regions across the globe from 1999 to 2003. The size of each regional segment corresponds to the relative contribution of each region to all collaborations produced worldwide. Tie widths at each end portray the relative contribution of collaborations between two regions to all collaborations associated with each individual region. (B) A circular plot of the same data from (A) except after normalization so that the collaborative output of each world region is the same. (C) A circular plot formatted in a similar manner to the plot in (A) except with data from 2009 to 2013. (D) A circular plot of the same data from (C) except after normalization so that the collaborative output of each region is the same. Regions are labeled as follows: Western Europe, WEu; Eastern Europe, EEu; Latin America, LAm; North America, NAm; Eastern Asia, EA; North Africa and Middle East, NAf&ME; South East Asia, SEA; Sub-Sahara, SS; South Asia, SA; Carribean, Ca.

Grouping nations by the UN human development (HD) index revealed that low HD countries have increased collaboration with medium HD countries over time at the expense of ties with high and very high HD countries. Moreover, internal collaborations increased and decreased for low HD and very high HD countries, respectively, while remaining minuscule in high and medium HD countries (Fig 6C and 6D). Tie formation organized by geopolitical regions also demonstrated the considerable and relatively uniform involvement of Western Europe in clinical research with other world regions (Fig 7). On the other hand, ties between either North America or East Asia (which includes research intensive countries such as China, Japan, and Korea) with non-European regions were diminutive. Notably, there was also a perceptible decline in the absolute and relative number of internal collaborations within Western Europe over the last decade.

**Fig 8:**
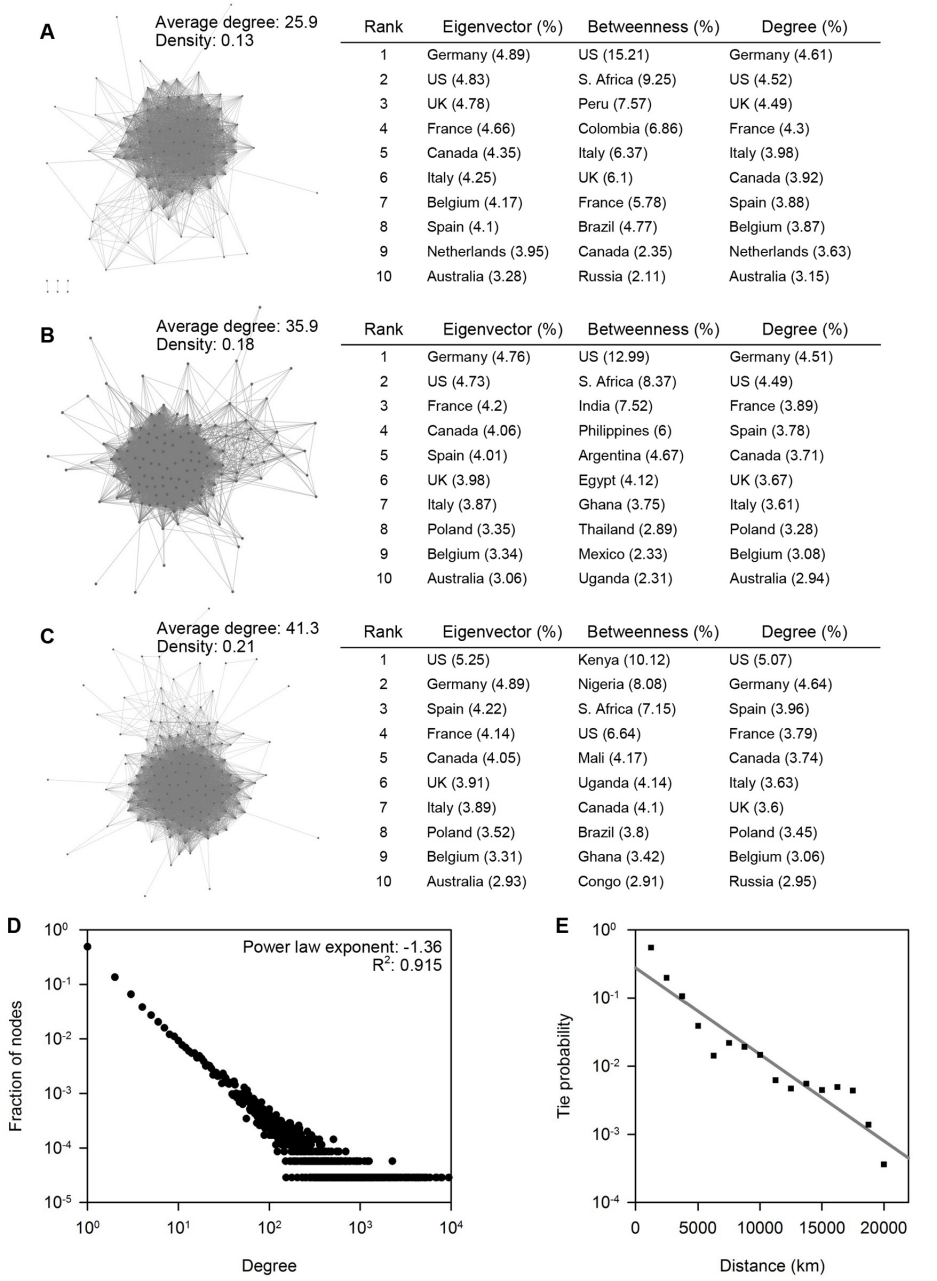
Properties of nation-level and city-level collaboration networks in clinical trials. (A), (B) and (C) The nation-level networks of multinational trials from 1999 to 2003, 2004 to 2008, and 2009 to 2013, respectively. Values of average network degrees and densities (number of edges over possible edges) are shown. Tables to the right in each panel display eigenvector, betweenness, and degree centrality measures (rescaled to percentages) for top ranking countries. (D) The degree distribution of the city-level clinical trial network. (E) The probability of two cities being involved in a clinical trial declines with increasing physical distance. Data points were binned at every 1250 kilometers.

Nation-level networks of clinical trials exhibited increased connectedness of participating nations over time worldwide, consistent with the fact that clinical trials have evolved to encompass more geopolitically discrete sites of enrollment (Fig 8A–8C). We also analyzed node-level characteristics to dissect the importance or relevance of individual countries. Specifically, we highlight eigenvector, betweenness, and degree centrality measures for top ranking countries over different time periods in Fig 8A, 8B and 8C. Eigenvector centrality assesses how well nodes are connected to other nodes with high Eigenvector centrality, giving greater weight to nodes that are linked to popular nodes. Thus, nations with high eigenvector centrality, such as Germany and the USA, are frequent collaborators with other popular nations involved in multinational research. Betweenness centrality assesses how many times a node is found along the shortest paths among all other nodes. Thus, nations with high betweenness centrality, such as South Africa and the USA, may be viewed as critical players in maintaining links between other countries or “cliques” and serving as “brokers” in the flow of information or collaborations. Degree centrality assesses the incidence of links pertaining to a node. Thus, nations with high degree centrality, such as Germany and the USA, can be regarded as being the most popular collaborating nations and are likely able to directly exchange or disperse information to the widest audience. Important features of the displayed results include the steady dominance of Western Europe, North America, and Australia among eigenvector and centrality measures, and the temporal flux of top ranked nations in betweenness centrality, suggesting a shift in the involvement of different world regions in clinical trials from South America (1999–2003), to South East Asia (2004–2008), and then to Africa (2009–2013). In fact, while European countries and the USA have the highest absolute number of links to other nations, Kenya, South Africa, and Nigeria have emerged as the most pivotal “bridges” in connecting countries within the global clinical research network.

To enhance spatial resolution of our network analyses, a city-level clinical trial network composed of ~40,000 nodes incorporating data from the last decade was also constructed. Importantly, the degree distribution of the city-level network was found to obey a power law which is observed in many social and biological networks and demonstrates the existence of well-connected cities serving as critical hubs in the collaborative network (Fig 8D) [22–25]. Networks characterized by power law degree distributions are also known as “scale-free” networks given that the connectivity of a random node varies widely, while in random networks most nodes have the same number of connections as the mean degree of the network which delineates the scale of the network. Scale-free structures in networks are remarkable given their association with distinctive growth and functional properties. For example, power-law degree distributions are hypothesized to arise from the growth of networks through preferential attachment of new nodes to well-established or popular nodes [22]. This suggests that collaboration of clinical sites principally arises from the outreach or the targeted involvement of established clinical research institutes. Scale-free networks are also tolerant of random failures or eliminations of most nodes, but very susceptible to the directed removal of well-connected nodes resulting in a rapid increase in the network diameter (the average length of the shortest path between any two nodes) and fragmentation of the entire network [26]. Thus, local or national policies governing highly connected clinical sites may have a disproportionally large impact on not only the most immediate ties, but also over even distant areas of the collaboration landscape.

We also determined the latitudes and longitudes of each city in order to determine the great circle distance (shortest physical distance between two points on a sphere) of each city-level network tie (Fig 8E). This allowed us to demonstrate that the probability of international ties in clinical trials diminishes over distances between cities, alluding to geography as a hindrance to collaborations.

## Discussion

The value of collaboration in medical research is unmistakable and work stemming from multinational teams has contributed to improvements in clinical practice and patient outcomes. Multinational clinical trials may also be advantageous for established nations and institutes in order to involve the participation of additional patient demographics and to potentially improve the health outcomes of other communities. For developing countries or less experienced institutes, international collaboration may be a critical source of additional expertise or finances and engender additional network contacts. The ongoing development of many drug and vaccine candidates in the USA and UK due to the 2014 Ebola virus epidemic in West Africa further exhibits the necessity for medical researchers to maintain a broad scope, particularly when available resources worldwide should be leveraged in instances where global health is threatened but may be controlled at a discrete source [27].

To our knowledge, this is the first study to elucidate trends in collaborations and the composition of transborder research teams pertaining to medical research involving human patients. Our results reveal a largely stagnant proportion of clinical trials involved in international collaborations spanning at least a decade, although there was also a propensity for clinical trials to involve an increasing number of nations over time. In particular, the dominating contribution of binary country relations in clinical research has waned due to the rapid emergence of larger scale collaborations. We also found that international clinical trials scaled with gross productivity in clinical research, and are associated with larger patient enrollments, industry funding, and specific medical specialties. Future studies are needed to examine whether such characteristics of international clinical trials are a prerequisite or consequence of the collaborative process. Clarifying the structure of national ties in clinical research may also pave way for future investigations on the effects of specific collaborations on trial success, clinical impact, and other possible consequences.

Our analysis did not rely on authorship data and thus includes trials that have not or may never be published, possibly providing a more inclusive analysis of clinical trials. However, our dataset is limited by the fact that not all clinical trials are necessarily registered (such as at Clinicaltrials.gov), although this is expected to be less likely for more recent years due to publication requirements in many journals and regional or institutional directives. Nevertheless, the wide scope of our study suggests that our findings are generalizable and may serve as a resource to guide research policies. For example, by illuminating patterns of international interactions in clinical trials, our study clarifies the relative isolation of geopolitical regions in research, indicating where targeted increases in the participation of investigators may lead to the inclusion of patients from underrepresented world regions within the medical community. Current such strategies likely underlie the observed flux in high betweenness countries.

Our assessment of the network topology of clinical trials also reveals how established institutes may be preferentially linked to emerging clinical sites. This connotes the tremendous influence of national, city, or institutional policies of “hubs” on the formation of new ties in international clinical trials and conceivably collaborative behaviors beyond local interactions. Thus, ambiguous or arbitrary regulations that effect the administration of clinical trials, industry relations, drug approvals, or the flow of medical information which make collaborations prohibitive, as recently exemplified in India, Russia, and other countries, may also have a delayed but rippling impact across the research network [28, 29]. Future investigations may substantiate this hypothesis by systematically analyzing whether changes in laws policing clinical research correspond with temporal trends in clinical trial output. Lastly, although advances in technology have enabled easier communication globally and likely decreased the cost of teamwork, our study indicates that physical distance represents an obstacle in clinical research collaborations. Distance between institutes may also encompass social, cultural, and economic factors, and further study is needed to comprehensively understand barriers in international clinical research.
